# Assembling Kidney Tissues from Cells: The Long Road from Organoids to Organs

**DOI:** 10.3389/fcell.2015.00070

**Published:** 2015-11-11

**Authors:** Krithika Hariharan, Andreas Kurtz, Kai M. Schmidt-Ott

**Affiliations:** ^1^Berlin-Brandenburg Center for Regenerative Therapies, Charité-Universitätsmedizin BerlinBerlin, Germany; ^2^College of Veterinary Medicine, Seoul National UniversitySeoul, South Korea; ^3^Department of Nephrology, Charité- UniversitätsmedizinBerlin, Germany; ^4^Max Delbrueck Center for Molecular Medicine in the Helmholtz AssociationBerlin, Germany

**Keywords:** pluripotent stem cells, organoids, stem cell differentiation, kidney development, vascularization

## Abstract

The field of regenerative medicine has witnessed significant advances that can pave the way to creating *de novo* organs. Organoids of brain, heart, intestine, liver, lung and also kidney have been developed by directed differentiation of pluripotent stem cells. While the success in producing tissue-specific units and organoids has been remarkable, the maintenance of an aggregation of such units *in vitro* is still a major challenge. While cell cultures are maintained by diffusion of oxygen and nutrients, three- dimensional *in vitro* organoids are generally limited in lifespan, size, and maturation due to the lack of a vascular system. Several groups have attempted to improve vascularization of organoids. Upon transplantation into a host, ramification of blood supply of host origin was observed within these organoids. Moreover, sustained circulation allows cells of an *in vitro* established renal organoid to mature and gain functionality in terms of absorption, secretion and filtration. Thus, the coordination of tissue differentiation and vascularization within developing organoids is an impending necessity to ensure survival, maturation, and functionality *in vitro* and tissue integration *in vivo*. In this review, we inquire how the foundation of circulation is laid down during the course of organogenesis, with special focus on the kidney. We will discuss whether nature offers a clue to assist the generation of a nephro-vascular unit that can attain functionality even prior to receiving external blood supply from a host. We revisit the steps that have been taken to induce nephrons and provide vascularity in lab grown tissues. We also discuss the possibilities offered by advancements in the field of vascular biology and developmental nephrology in order to achieve the long-term goal of producing transplantable kidneys *in vitro*.

## Introduction

While our knowledge on the structure and function of mature organs is mostly derived from animal models, human cadavers, or biopsies, the information we have on embryonic organ development is largely derived from various model organisms and from technologies that study human embryogenesis *in vitro*. In 1944, the first human egg was fertilized *in vitro* (Rock and Menkin, 1944). Edwards and Steptoe implanted a fertilized egg into a woman's uterus in 1977 (Steptoe and Edwards, 1978) giving the world its first test tube baby. These scientists were also paving the way for a closer observation of human embryonic development. Edwards and his team had worked on growing human embryos *in vitro*, until the blastocyst stage (Edwards et al., 1981) before re-implanting the embryo into the uterus. Knowing that *in vitro* culture was a suitable interim step in producing a viable organism spurred the goal of replacing damaged organs by transplanting organoids produced *in vitro*. Key advances in this field included the derivation of human embryonic stem cells (hESC) from the inner cell mass of normal human blastocysts (Thomson et al., 1998) and the generation of induced pluripotent stem cells (iPSC) (Takahashi and Yamanaka, 2006; Takahashi et al., 2007). These cells demonstrate evidence of stable developmental potential even after prolonged culture forming derivatives of all three embryonic germ layers from the progeny of a single cell. A huge advantage of iPSC over ESC, is the possibility to have pluripotent stem cells from every individual, providing an opportunity to study and repair genetic disorders at a personal level. Pluripotency has been exploited to recapitulate many embryonic processes *in vitro*, including primitive streak formation, neural tube induction, and trophoblast formation, extending to the generation of functioning neurons, beating cardio-myocytes, and insulin producing beta cells of the pancreas. Major achievements in the field of stem cell biology have been the derivation of a multitude of differentiated cell types morphologically similar to their terminally differentiated counterparts *in vivo*, but functionally immature *in vitro*, that have been intended to be used as regenerative cell sources in degenerative disease conditions. However, in order to model and study human disease, and to subsequently test and develop drugs, functionality of terminally differentiated cells has to be achieved.

Mammalian cells, as part of multicellular organisms, function in tissue units that contain several types of cells, which together form an organ. The fact that many ESC and iPSC—derived cell types display a mostly immature, fetal phenotype suggests that multicellularity is also a prerequisite for terminal differentiation. Nevertheless, the ability to derive specific cell types of any organ from PSCs has provided the possibility of constructing functional units of an organ, containing distinct cell types. Several groups pioneered the development of tissues *in vitro* from PSCs, including 3D cortical neuro-epithelium with up to 6 layers of neurons (Eiraku et al., 2008), intestinal (Spence et al., 2011), retinal, liver, inner ear, and kidney organoids (Eiraku et al., 2011; Nakano et al., 2012; Koehler et al., 2013; Takebe et al., 2013; Takasato et al., 2014) These novel findings prompted a redefinition of the term “organoid” as a collection of organ-specific cell types that develop from stem cells or organ progenitors and self-organize through cell sorting and spatially restricted lineage commitment in a manner similar to the situation *in vivo* (Lancaster and Knoblich, 2014). Despite all the progress, no functional nephron or liver acini unit has been generated *in vitro*. In this review, we will revisit methods to differentiate tissue-specific cells from pluripotent stem cells, to achieve 3-dimensional tissue modeling, and discuss current efforts to enhance vascularization of organoids *in vitro* and *in vivo*.

## Mirroring organogenesis *in vitro*

### Kidney-specific cell differentiation from pluripotent stem cells

Humans are triploblastic and thus, develop a tri-laminar embryo upon gastrulation, consisting of ectoderm, endoderm, and mesoderm. Several groups have cultured PSCs in a feeder free matrix-coated surface and promoted differentiation by adding growth factors and inhibitors to this 2D system, while other groups applied inductive media to a 3D system, after the generation of a sphere of PSC cells known as an embryoid body (EB). Protocols applying defined time periods and concentrations of exposure to inductive agents have allowed for the differentiation of endo-, ecto-, or mesodermal lineages and specific cell types. For example, activin signaling leads to 80% efficient induction of SOX17^+^/GSC^+^/FOXA2^+^/MIXL1^+^ definitive endoderm in hESC cultures after 5 days of differentiation in 100 ng/ml activin A (D'Amour et al., 2005). In another seminal study, sequential treatment of high-density undifferentiated monolayer hESC cultures with activin A for 24 h and bone morphogenetic protein 4 (BMP4) for 4 days consistently yielded >30% cardiomyocytes (Laflamme et al., 2007). For neural induction of hESCs, the growth factors insulin, epidermal growth factor (EGF) and basic fibroblast growth factor (bFGF) were added in a chemically defined medium giving rise to up to 90% PAX6^+^ neural progenitors (Joannides et al., 2007). These studies have proved that while a spontaneously differentiating mass of PSC can generate varying proportions of endo- and ecto- and mesoderm, targeting signaling pathways prominent in the development of an organ of interest in a temporal fashion can promote that particular lineage. Over the years, researchers have tested various combinations of cell signaling modifiers to achieve enrichment of tissue-specific cell types in a heterogenous population of differentiated PSC progeny (reviewed by Murry and Keller, 2008). Moreover, the level of enrichment varies with the iPSC or ESC used. iPSC that are reprogrammed from a somatic cell type originating from an organ and are differentiated to a cell type of the same organ, show higher efficiencies owing to epigenetic memory of the source (Hiler et al., 2015). This highlights the need for standardization of protocols with certain PSC lines for certain lineages.

Unlike other organs, few protocols have been established for the kidney. The kidney exhibits a remarkable architectural complexity coupled with the presence of at least 26 different specialized cells (Al-Awqati and Oliver, 2002). The kidney is mesodermal in origin, arising from the intermediate mesoderm (IM). Arising from IM, the ureteric bud (UB) interacts with an adjacent cell population, the metanephric mesenchyme (MM). Through reciprocal interactions, the ureteric bud will generate the ureteric tree giving rise to the ureter, renal pelvis and collecting ducts, while progenitors within the metanephric mesenchyme will generate nephrons (Grobstein, 1953; Saxén and Sariola, 1987). Based on earlier studies that highlighted important molecules and pathways that drive mesodermal and nephron differentiation in model organisms, initial studies of differentiation toward the renal lineage were performed on mouse ESC EBs treated with media containing serum together with multiple combinations of factors including activin A, BMP4, BMP7, retinoic acid (RA), leukemia inhibiting factor (LIF), and glial-derived neurotrophic factor (GDNF) or UB-derived conditioned media. These protocols led to the generation of cells expressing markers of differentiation, e.g., Pax2 (kidney tubules), Aquaporin-2 (collecting duct principal cells), Wt1 (metanephric mesenchyme and podocytes), or Ksp-Cadherin (distal nephron tubules), within EBs, which provided evidence of successful renal lineage induction (Kobayashi et al., 2005; Bruce et al., 2007; Vigneau et al., 2007; Morizane et al., 2009; Ren et al., 2010; Nishikawa et al., 2012). Although a renal identity was achieved in the examples mentioned before, the desired cell types could not be isolated owing to low and varying frequencies of occurrence and their functionality was not demonstrated.

Meanwhile, genetic lineage tracing demonstrated that the induced Six2-expressing cap mesenchyme represents a nephron progenitor population that gives rise to all cell types of the nephron (Kobayashi et al., 2008). Also, the intermediate mesodermal origin of kidney cells was confirmed when Mugford et al., used molecular fate mapping to demonstrate that the majority of cell types within the metanephric kidney arise from an Osr1-expressing population within the intermediate mesoderm (Mugford et al., 2008). These new findings were considered in differentiation protocols to distinguish the exact mesodermal cell sub-type required to enrich cultures of PSC-derived renal progeny. For instance, Mae et al developed a robust protocol using activin A and Wnt-agonist CHIR99021 for 2 days and sequential treatment with BMP7 and CHIR99021 for 8 days to obtain 90% OSR1^+^ cells (Mae et al., 2013). Despite such an efficient protocol, the dependence on OSR1 as a population identifier created ambiguity, since even though OSR1 is expressed in the intermediate mesoderm, it is also expressed in the earlier mesoderm prior its subdivision into paraxial and intermediate domains (Guillaume et al., 2009). Moreover, OSR1 is expressed in both the intermediate mesoderm and lateral plate (James and Schultheiss, 2003; Wilm et al., 2004).

Additional protocols were developed to induce different cell types within the nephron. Human PSC derived podocytes expressing podocin, nephrin, and synaptopodin were generated from EBs using treatment with Activin A, RA, and BMP7 and plating on gelatin (Song et al., 2012). In another study, ~30% AQP1^+^ proximal tubule cells were obtained by treating a monolayer PSC culture with media containing RA, BMP2, and BMP7 for 20 days (Narayanan et al., 2013). Recent reports demonstrated stepwise induction of UB and/or MM through systematic induction of primitive streak alone, followed by intermediate mesodermal specification (Xia et al., 2013, 2014; Lam et al., 2014; Taguchi et al., 2014; Takasato et al., 2014). These studies performed thorough characterization of cell types obtained at every stage, focusing on obtaining PAX2^+^ GATA3^+^ LHX1^+^ UB cells (Xia et al., 2013) and SIX2^+^ PAX2^+^ OSR1^+−^GDNF^+^ HOX11^+^ WT1^+^ MM cells (Taguchi et al., 2014). Takasato et al. were able to generate UB and MM between 14 and 18 days, whereas Lam et al generated SIX2^+^ SALL1^+^ WT1^+^ cap mesenchyme by 8 days and showed the potency of PAX2^+^ LHX1^+^ imtermediate mesodermal cells to generate tubule structures that express Lotus lectin and Ksp-Cadherin after 9 days of differentiation (Lam et al., 2014; Takasato et al., 2014).

Together, these important studies provided proof of principle that most if not all components of the kidney can be induced from PCS. The PSC-derived UB cells can be utilized to generate a ureteric tree or PSC-derived MM cells can be coaxed to produce S-shaped bodies that undergo proximal distal patterning initiating tubulogenesis, giving rise to fetal nephrons in culture. It would also be interesting to use cells derived from the Xia protocol to generate UB and Taguchi protocol to obtain MM and investigate if they interact in a similar manner as these cell types do *in vivo*. Since most kidney diseases involve the damage and loss of podocytes or hypertrophy of tubular epithelial cells, these cell types have a high priority of being derived. On the other hand, the big picture of nephron reconstruction requires other specialized cells including mesangial cells, glomerular endothelial cells, epithelial cells of the loop of Henle, principal cells and intercalated cells that have not yet been procured from PSCs. Induction of terminal differentiation, recapitulation of the architectural context and building of functional nephrons of the kidney are key challenges to be mastered.

### Morphogenesis and patterning

The development of an organism involves not only differentiation of cells, but also their morphogenesis and appropriate patterning to form the architectural context of tissues and organs (Gilbert, 2000). Essentially, cells need to communicate with each other and their microenvironment, by means of growth factors, morphogens, cell adhesion molecules, and mechanoreceptors. The elegant experiments of Holtfreter and Townes studying Xenopus embryo re-aggregation patterns and organization indicated that a whole system of attraction and repulsion phenomena operates between various cell types during development and that information on this system will yield valuable information concerning the segregation of tissues during organogenesis. Holtfreter used the term “tissue affinity” (Gewebeaffinität) to describe this force (Holtfreter, 1939, 1944; Townes and Holtfreter, 1955). Steinberg's differential adhesion hypothesis supported the ideas of Holtfreter with thermodynamic principles (Steinberg, 1970) providing a partial explanation of the self-organizing properties of tissues. Embryonic tissue re-aggregation and adult organ-derived cells have demonstrated this property, e.g., in liver, lung, eye, brain, and thyroid gland (Weiss and Taylor, 1960; Grover, 1961a,b; Ishii, 1966; Hilfer et al., 1968; Stefanelli et al., 1977). The observation of such tissue affinity and self-assembly among PSC-derived progeny flags the success of recapitulating natural embryological behavior *in vitro*.

Toward this extent, one of the first reports of such a phenomenon in mouse PSC-differentiated cells came from the Sasai lab. The appearance of multi-layered cortical neuro-epithelium in serum-free embryoid body-like aggregates in suspension culture, showed a similar pattern as observed during development. This remarkable finding could also be reproduced in hESCs (Eiraku et al., 2008). Further examples of self-organizing tissue morphogenesis were the generation of optic cups from mouse ESCs (Eiraku et al., 2011) and of neural retina from human ESCs (Nakano et al., 2012). Lancaster et al., developed 3-dimensional self-organizing cerebral organoids from human ESCs, which develop distinct interconnected brain regions (Lancaster et al., 2013). Spence et al. developed self-organizing intestinal organoids from human ESCs, which recapitulated cell types and the stem cell compartment of the intestine (Spence et al., 2011).

Partial self-organization of mouse embryonic kidney cells upon their re-aggregation after dissociation was first achieved by Unbekandt and Davies (2010). The novelty of this system was the introduction of a ROCK inhibitor, which prevented the dissociation-induced apoptosis within single cell suspensions and facilitated a significant recovery of re-aggregated tissues. The Unbekant re-aggregation method has since then been used as a test system to check the capacity of cells (e.g., PSC-derived cells) to integrate into forming tubules or glomeruli of the mouse nephron. While this method proves the property of test cells to contribute to kidney formation, it cannot provide proof of self-organization of PSC-derived renal progenitors. To this end, cases of kidney organogenesis from PSC have been reported by Takasato et al., where 18 days of differentiation of PSC seeded initially on matrigel, develop an ECAD^+^ ureteric epithelium surrounded by clumps of SIX2^+^ WT1^+^ PAX2^+^ MM cells or JAG1^+^ CDH6^+^ renal vesicles (Takasato et al., 2014). Lam et al. also observed appearance of tubule-like structures positive for Lotus lectin (a proximal tubule marker) from SIX2+ cap mesenchyme cell cultures, obtained on day 7 of their differentiation procedure, upon treatment with CHIR99021 (Lam et al., 2014). This was reminiscent of induced metanephric mesenchyme that responds to Wnt signals to undergo mesenchymal-epithelial transition and form renal vesicles *in vivo* (Park et al., 2007; Schmidt-Ott et al., 2007). Meanwhile, the group of Nishinakamura had also obtained evidence of a slightly different nature. They used PSC in the form of EBs for a differentiation protocol that took 8.5 days in mouse ESCs and 14 days in human iPSCs, resulting in SIX2^+^ WT1^+^ SALL1^+^ PAX2^+^ MM cells that could give rise to tubules and podocytes when induced by mouse embryonic spinal cord (Taguchi et al., 2014). These studies are evidence that a systematic mirroring of embryonic kidney development in PSC derivatives can lead to the formation of organo-typical structures, as summarized in Table 1. This brings us a step closer to develop nephrons *in vitro*, as these structures can be coaxed under concentration gradients of growth factors to boost tubulogeneis. Yet, there is clearly a need to enhance protocols to achieve full maturation of nephrons that have on one hand a filtering unit and on the other hand an optimal spatial orientation of tubules and trigger their functionality in terms of electrolyte transport.

**Table 1:** **Status of lab-grown kidney organoids**.

**Cell source**	**Description**	**References**
***IN VITRO***		
Embryonic cells	Suspension culture of dissociated-reaggregated chick mesonephric cells	Moscona and Moscona, 1952
	Culture at air–medium interface of dissociated- reaggregated mouse metanephric cells	Unbekandt and Davies, 2010
Adult cells	Collagen matrix embedded murine and human renal cells	Joraku et al., 2009; Guimaraes-Souza et al., 2012
PSC- derived cells	2-dimensional culture of 18 day differentiated hPSC	Takasato et al., 2014
	14-day differentiated EBs induced with mouse embryonic spinal cord	Taguchi et al., 2014
***IN VIVO***		
Embyonic cells	Mouse metanephric cells cultured on CAM of avian embryos	Preminger et al., 1980
	Rat metanephroic cells transplanted in the omentum of a rat	Hammerman, 2002
	Mouse metanephric cells cultivated in mouse lymph node	Francipane and Lagasse, 2015
PSC-derived cells	Mouse embryonic spinal cord-induced EBs transplanted under the kidney capsule of mice	Taguchi et al., 2014
	Sall1- deficient mouse blastocyst, complemented with wildtype mouse PSCs	Usui et al., 2012

A niche for organogenesis was created in post-blastocyst mutant mouse embryos that are genetically precluded from developing a particular organ by Kobayashi et al. The group generated a chimeric animal where injected PSC-derived cells colonized this developmental niche and compensated for the developmental defect to form a donor-induced organ *in vivo*. They injected rat wild-type PSCs into *Pdx1*^−∕−^ mouse blastocysts, generating normally functioning rat pancreas in *Pdx1*^−∕−^ mice. These data constituted proof of principle for interspecific blastocyst complementation and for generation of organs derived from donor PSCs using a xenogenic environment *in vivo* (Kobayashi et al., 2010). On similar lines, the same group injected mouse PSCs in *Sall1*-deficient mice, since *Sall1* is expressed in the metanephric mesenchyme–derived structures in the developing kidney. The kidneys of *Sall1*^−∕−^ chimeric mice were almost entirely composed of EGFP-marked mouse iPSC-derived cells. These iPSC-derived kidneys of *Sall1*^−∕−^ chimeric mice were grossly normal in shape and size. Bladders of these mice were filled with urine, indicating that the iPS-derived kidneys were functional and appropriately connected to the lower urinary tract (Usui et al., 2012). It can be anticipated that human organs can similarly be developed in animal hosts.

## Revisiting renal development—maturation of nephrons

Kidney organoids obtained *in vitro* are comparable to E12.5–E13.5 metanephric mouse kidneys, where in, the ureteric tree has branched and the cap mesenchyme undergoes mesenchymal-to-epithelial transition into renal vesicles, which elongate and undergo patterning to form comma-shaped and S-shaped bodies that are scattered in the cortex. In parallel, the renal stroma, derived from Foxd1^+^ cells and Flk^+^ cell derived vasculature, are also contributing to development of kidney architecture (Hatini et al., 1996; Robert et al., 1996; Abrahamson et al., 1998). Formation of renal vasculature is a combination of angiogenesis and vasculogenesis. The lateral branch of the aorta invades the kidney at E12.5 and becomes the renal artery that has 3–4 branches by E13.5. Around E17.5, the arterial tree extends until the cortex due to strong VEGFA signals from developing podocytes in the glomerular zone, leading to the formation of afferent arterioles. Although it has been observed that at around E13-14, endothelial cells migrate into the cleft of glomeruli to form a capillary network, the source of these cells remains elusive (Herzlinger and Hurtado, 2014). Lineage tracing of Tie1/LacZ E11 metanephroi transplanted into a nephrogenic cortex has shown that endothelial precursors exist before the onset of nephrogenesis since the donor tissue showed transgene-expression in glomerular capillary loops (Loughna et al., 1997). A cKIT^+^ cell population originating from the aorta-gonad-mesonephros hemangioblasts has also been observed during E10.5–E11.5 that are distinct from Foxd1+ stromal cells (Schmidt-Ott et al., 2006), the fate of these cells has not been examined. Foxd1+ stromal derivatives differentiate into peritubular capillary endothelial cells that can be observed around E18.5 (Sims-Lucas et al., 2013) and also secrete factors required for normal nephron and vascular differentiation (Das et al., 2013; Hum et al., 2014).

Rymer et al. performed *in utero* embryonic intra-cardiac injection of tomato lectin (TL) to label perfused blood vessels at different stages of development (E11.5–E17). They found that perfused blood vessels were closely linked to mature nephron structures, while the nephrogenic zone did not show evidence of blood flow. This suggests that terminal differentiation is linked to an oxygen-rich environment, while multipotent nephron progenitors exist under low oxygen conditions (Rymer et al., 2014). This finding is potentially important with respect to tissue engineering, since strategies to establish blood flow and oxygenation within organoids may provide a critical step toward generating mature renal tissues.

## Bringing blood into an organoid—vascularization strategies

PSC-derived tissue specific cell types irrespective of being generated in a 2- or 3-dimensional culture are functionally immature despite having morphological properties of the desired cell type. Essential functions of the kidney, including glomerular filtration, tubular reabsorption, or hormone secretion, essentially dependent on the presence of a vasculature. In addition, the diffusion limit of oxygen in organ cultures is around 150 μm, while the organoids that are currently being developed range from 1 to 2 mm, raising an issue of oxygen deprivation in the core regions gradually leading to tissue decay.

Learning from embryonic vascular development, each organ has progenitors distributed in the primordia that develop into the vasculature of the respective organ. Growth factors acting on these progenitors, including VEGF (Tufro et al., 1999), stem cell factor (SCF) (Schmidt-Ott et al., 2006), and Ang1 (Loughna et al., 1997; Woolf et al., 2009) can be supplemented into culture media to enrich the differentiated progeny. Xinaris et al. used 5-day-organoids derived from E11.5 mouse kidney, dissociated into single cells, and used a protocol involving VEGF treatment and transplantation of these cells under the kidney capsule of athymic rats. This facilitated vascularization with blood vessels from donor (mouse) origin, *in vivo* viability for at least 3 weeks, and glomerular maturation characterized by formation of capillary walls with fenestrated endothelium, podocytes with foot processes and slit diaphragms. Moreover, FITC-conjugated albumin injections were recovered in proximal tubules suggesting successful glomerular filtration and tubular reabsorption in these allografts (Xinaris et al., 2012).

Another approach that could enhance vascularization of organoids that are considerably thick is *in vitro* addition of vascular and perivascular cells. Formation of micro-capillary like structures by endothelial cells prior to transplantation accelerates anastomosis with host vasculature. The first report of *in vitro* pre-vascularized tissues was the generation of a skin equivalent by culturing dermal fibroblasts, keratinocytes and human umblical veil endothelial cells (HUVECs) (Black et al., 1998; Tonello et al., 2003). Stevens et al constructed “tri-cell” cardiac patches containing hESC-derived cardiomyocytes, HUVECs, and mouse embryonic fibroblasts (MEFs) in 1:1:0.5 ratios, respectively (cardio-HUVEC-MEF patches). In sharp contrast to cardio-HUVEC patches, addition of MEFs to human cardiomyocytes and HUVECs resulted in the formation of human CD31-positive endothelial cell networks that morphologically resembled a vascular plexus (Stevens et al., 2009). Similar success has been reported in generating a human liver bud where PSC-derived hepatic progenitors, human mesenchymal stromal cells(hMSC) and HUVECs were co-cultured *in vitro* prior to cranial transplantation in immune-deficient mice (Takebe et al., 2013). As a development to the previous study, Takebe et al. also developed kidney buds by substituting PSC-derived hepatic progenitors with E13.5 kidney derived dissociated cells that could form glomerular like micro-tissues after 8 days of transplantation followed by quick vascularization (Takebe et al., 2015).

A step closer toward a vascularized organoid is the use of scaffolds that are pre-vascularized with HUVECs with or without perivascular cells, followed by seeding of dissociated cells of the organoid or just PSC-derived sorted progenitors. Acellular porcine or rodent kidney matrices have been repopulated with HUVECs lining vessel walls and fetal kidney cells in tubular and glomerular compartments; orthotopic transplantation of the reseeded kidney in single nephrectomized rats showing blood flow through the vasculature and urine production (Song et al., 2013). Miller et al. printed rigid 3D filament networks of carbohydrate glass in the engineered tissue to generate cylindrical networks that can be lined by endothelial cells and perfused, offers an independent extravascular tissue. Initially, a mixture of glucose, sucrose, and dextran was developed such that it formed a glass when cooled and optically transparent such that it could be used in conjunction with photo-cross-linkable materials. The carbohydrate glass was then extruded through a heated syringe into an interconnected microfluidic vascular network allowed to cool, and a scaffold was cast around it in a mixture of mouse fibroblast cells and photo-cross-linkable ECM pre-polymer. When immersed in water, the carbohydrate quickly dissolved, leaving a hollow network in its place (Miller et al., 2012). Bio-printing and micro-fabrication of cells embedded in hydrogel in a pattern generated from knowledge of renal architecture is an emerging technology that is still in its infancy. Currently, acellular adult kidneys and bio-printing are methods that may provide architectural features similar to that of an adult organ, and hold a promising future for creating vascularized transplantable organs *in vitro*. Current limitations of bioprinting are dimensional, with minimal sizes approaching 30–50 μm, and the availability of adequate materials. Extracellular matrix printing seems to provide some benefit over other polymers in terms of cell survival and differentiation. Its combination with shape controllable polymers, for example to print preformed scaffolds that are then filled with matrix containing cells or cell clusters will perhaps lead to readily generated 3-dimensional and properly tubulized tissues. What remains to be figured out is which cell type can be chosen to repopulate such a pre-vascularized-scaffold. Among various other candidates, would several PSC-derived organoids occupy the matrix and develop a nephron-vascular system or is it sufficient to use a cocktail of PSC-derived terminal cell types and allow them to anchor to the scaffold and attain function?

The simplest method to vascularize the kidney organoid would involve connection to an extrinsic vascular supply. This was first demonstrated by Sariola et al., when they grafted mouse embryonic kidney explants on to chorio-allantoic-membrane (CAM) of a quail and maintained them for 5–10 days. The grafted kidney gave rise to well- developed convoluted secretory tubules and highly branched collecting ducts. Definitive glomeruli were also identified in these grafts by the presence of efferent tubules, visceral and parietal epithelium, capillary tuft, and Bowman's space. These CAM grafts appeared histologically comparable to the 14- 15 -day *in vivo* embryonic metanephros (Preminger et al., 1980). Following this example, Taguchi et al placed mouse-PSC-derived MM cell mass induced by mouse spinal cord beneath the kidney capsule of immunodeficient mice. When harvested after a week, tubulogenesis and glomerular formation was seen as expected and around 84.6% of the glomeruli showed integration of vessels from the host. This demonstrates maturation of the induced EB in presence of a vascular supply (Taguchi et al., 2014).

Ectopic organogenesis has been extended to several sites *in vivo*, since the kidney capsule restricts space availability for the maturating renal primordia. Transplantation of E15 rat metanephroi into omentum of non-immunosuppressed adult rats resulted in a normal kidney structure and function following uretero-uterostomy, surviving as long as 32 weeks (Hammerman, 2002). A recent study by the Lagasse group has reported the successful organogenesis of the kidney using the mouse lymph node as a developmental niche. Some of their findings include the survival of neo-kidneys until the 12th week of transplantation, engraftment and maturation of metanephroi although fluid waste accumulation resulted in graft degeneration. Moreover, nephrectomy of the mice resulted in accelerated organogenesis suggesting a compensatory response (Francipane and Lagasse, 2015). To determine whether the fetal kidney could provide a suitable niche for MSCs to generate renal tissue, Yokote and Yokoo (2013) injected GDNF-expressing human MSCs into rodent whole-embryo cultures at the point of budding. MSC were shown to integrate into the developing fetal kidney forming a chimeric organ leading to a possibility that depletion of animal cells in this chimeric kidney after transplantation into the omentum of human patients would allow further development of the kidney only from human cells (Yokoo and Kawamura, 2009; Yokote and Yokoo, 2013). The result that we aim for in any case, is a kidney that when perfused with blood/ equivalent of body fluids can filter larger proteins and reabsorb electrolytes to secrete a urine concentrate into a functional ureter. Further molecular and cellular investigations of the afore-mentioned experiments of *in vivo* vascularization of kidney organoids (listed in Table 2) can provide valuable lessons on how the vascular networks are established and can be transferred to *in vitro* experiments conducted on scaffolds, increasing our chances to develop a nephron-vascular unit.

**Table 2:** **Strategies to develop a nephro-vascular unit**.

**Strategy**	**Description**	**References**
Growth factor induction	VEGF, SCF, Ang-1	Loughna et al., 1997; Tufro et al., 1999; Schmidt-Ott et al., 2006; Woolf et al., 2009
Co-culture with peri-vascular cell types	Fibroblasts and endothelial cells	Black et al., 1998; Tonello et al., 2003
	Mesenchymal stromal cells and endothelial cells	Takebe et al., 2013, 2015
Providing architectural and mechanical support	Endothelialization followed by repopulation with kidney cells of • an acellular organ matrix • 3D printed polymer-based network Suitable for perfusion through endothelial channels	Nakayama et al., 2010; Miller et al., 2012; Song et al., 2013
***IN VIVO***		
Providing a niche for ectopic organogenesis	CAM	Preminger et al., 1980
	Beneath the kidney capsule of a rodent	Taguchi et al., 2014
	Omentum of a rodent	Hammerman, 2002
	Rodent lymph node	Francipane and Lagasse, 2015

## Outlook

The options for generating vascularized tissues suitable for *in vitro* assays or for regenerative medicine have increased greatly in the last decade. Vascularization is achievable *in vitro* by means of preformed natural or artificial printed or casted scaffolds or pre-established endothelialized networks, which can then be populated with tissue cells, depending on the application. The populating cells may lead to further remodeling and capillary formation, which could be directed using switchable expression of angiogenic factors. Alternatively, supplying tissues, cells or organoids undergoing morphogenesis with vascular or lymphatic precursor cells may be useful to enable the tissue to autonomously generate vascularity or to allow faster vascularization post transplantation. The perfusion of the vascular system *in vitro* will become feasible with advanced microfluidics devices and bioreactors for larger organs.

In addition, vascularization of preformed avascular organoids is achievable *in vivo*, by transplantation either ectopically or at the organ specific target site. The aggregation of pre-formed smaller functional tissue units or organoids may be a technique to build larger organs. These smaller units could be either avascular or preconditioned with endothelial cells or suitable scaffolds to quickly vascularize the larger aggregates. The provision of larger vessel adapters to allow direct connectivity to the host's vascular net will provide blood supply early after transplantation. Finally, the generation of embryonic or adult human organs *in vivo* would allow complete vascularization after transplantation to environmental niches that permit continuing organogenesis. These niches could be provided *in vitro* and *in vivo*. Based on this approach, growth of pre-vascular fetal human kidneys in transgenic kidney deficient mice may be used to generate kidney primordia for transplantation and vascularization in adult human hosts. The establishment of a fully humanized kidney *in vivo*, including human derived vasculature and innervation may also be achieved using hMSC in an animal model as demonstrated by Yokote et al. (2015). Similarly, adult kidneys could be generated in donor animals that promote development of fully humanized organs and transplanted or used in perfusion systems.

Striking advances have also been achieved regarding the toolbox for generating human cells that are useful to generate tissue specific organoids. This includes the establishment of well characterized adult and fetal tissue derived primary differentiated and precursor cells, as well as of PSC-derived endothelial, lymphangiogenic, and organ specific cells. Endothelial cells and vasculatures have tissue-specific characteristics, for example in the kidney glomerulus they are highly fenestrated and differentiate in close association with podocytes. Whether iPSC-derived endothelial cells are able to acquire the tissue specific phenotype needs to be investigated. The design of cells with tailored function is another option where the basic technologies are already known. It can be expected that design of cells will advance to the tissue and organoid level including cells with vascularizing functions.

Applications of organoids, which model function and structure of the kidney, are not restricted to understanding human kidney development. They are applicable to study the pathogenesis of genetic diseases as patient –derived PSC can be used for organoid generation. These are equally applicable for drug testing as PSC can be stratified according to patient cohorts and organoids functionally characterized *in vitro* as well as *in vivo* post grafting. Finally, organoids may be applicable in regenerative medicine to replace or repair functional tissue units.

### Conflict of interest statement

The authors declare that the research was conducted in the absence of any commercial or financial relationships that could be construed as a potential conflict of interest.
